# Phytochemicals That Influence Gut Microbiota as Prophylactics and for the Treatment of Obesity and Inflammatory Diseases

**DOI:** 10.1155/2018/9734845

**Published:** 2018-03-26

**Authors:** Lucrecia Carrera-Quintanar, Rocío I. López Roa, Saray Quintero-Fabián, Marina A. Sánchez-Sánchez, Barbara Vizmanos, Daniel Ortuño-Sahagún

**Affiliations:** ^1^Universidad de Guadalajara, Laboratorio de Ciencias de los Alimentos, Departamento de Reproducción Humana, Crecimiento y Desarrollo Infantil, CUCS, Guadalajara, JAL, Mexico; ^2^Universidad de Guadalajara, Laboratorio de Investigación y Desarrollo Farmacéutico, Departamento de Farmacobiología, CUCEI, Guadalajara, JAL, Mexico; ^3^Universidad Nacional Autónoma de México, Instituto Nacional de Pediatría, Unidad de Genética de la Nutrición, Instituto de Investigaciones Biomédicas, Mexico City, Mexico; ^4^Universidad de Guadalajara, Laboratorio de Neuroinmunobiología Molecular, Instituto de Investigación en Ciencias Biomédicas (IICB), CUCS, Guadalajara, JAL, Mexico; ^5^Universidad de Guadalajara, Laboratorio de Evaluación del Estado Nutricio, Departamento de Reproducción Humana, Crecimiento y Desarrollo Infantil, CUCS, Guadalajara, JAL, Mexico

## Abstract

Gut microbiota (GM) plays several crucial roles in host physiology and influences several relevant functions. In more than one respect, it can be said that you “feed your microbiota and are fed by it.” GM diversity is affected by diet and influences metabolic and immune functions of the host's physiology. Consequently, an imbalance of GM, or dysbiosis, may be the cause or at least may lead to the progression of various pathologies such as infectious diseases, gastrointestinal cancers, inflammatory bowel disease, and even obesity and diabetes. Therefore, GM is an appropriate target for nutritional interventions to improve health. For this reason, phytochemicals that can influence GM have recently been studied as adjuvants for the treatment of obesity and inflammatory diseases. Phytochemicals include prebiotics and probiotics, as well as several chemical compounds such as polyphenols and derivatives, carotenoids, and thiosulfates. The largest group of these comprises polyphenols, which can be subclassified into four main groups: flavonoids (including eight subgroups), phenolic acids (such as curcumin), stilbenoids (such as resveratrol), and lignans. Consequently, in this review, we will present, organize, and discuss the most recent evidence indicating a relationship between the effects of different phytochemicals on GM that affect obesity and/or inflammation, focusing on the effect of approximately 40 different phytochemical compounds that have been chemically identified and that constitute some natural reservoir, such as potential prophylactics, as candidates for the treatment of obesity and inflammatory diseases.

## 1. Introduction

Obesity is a chronic state of low-grade inflammation constituting a well-known risk factor for multiple pathological conditions, including metabolic syndrome and insulin resistance [1], and it has also been implicated as a proactive factor and associated with a nonfavorable disease course of chronic autoimmune inflammatory disorders, such as multiple sclerosis (MS) [2]. Several studies over the last decade report interest in fermentation products from gut microbiota (GM) in the control of obesity and related metabolic disorders [3]. GM denotes an entire ecosystem inhabiting each organism, thus constituting a “superorganism” [4]. GM plays several crucial roles in host physiology and influences several relevant functions: it harvests energy from indigestible food, influences fatty acid oxidation, fasting, bile acid production, satiety, and lipogenesis, and even influences innate immunity (reviewed in [3]). In more than one respect, we are able to establish that you “feed your microbiota and are fed by it.” GM provides signals that promote the production of cytokines, leading to the maturation of immune cells modulating the normal development of immune functions of the host immune system [5, 6]. Consequently, an imbalance of GM, or dysbiosis, can be the cause or at least lead to the progression of several pathologies such as infectious diseases, gastrointestinal cancers, cardiovascular disease, inflammatory bowel disease, and even obesity and diabetes [7, 8]. Additionally, a pathological state can cause an imbalance in this microbial ecosystem. For instance, a dysfunction of the innate immune system may be one of the factors that favor metabolic diseases through alteration of the GM [9].

In terms of immune response, the immune system recognizes conserved structural motifs of microbes, called PAMPs (pathogen-associated molecular patterns), by mean of toll-like receptors (TLR), which are expressed in the membrane of sentinel cells [10]. This interaction induces immune responses against microbes through the activation of inflammatory signaling pathways. Therefore, GM, which interacts with epithelial TLR, critically influences immune homeostasis [9]. Although the complete etiology of inflammatory diseases remains unknown, intestinal gut dysbiosis has been associated with a variety of neonatal and children's diseases [4], in which chronic intestinal inflammation and mucosal damage derives from alteration of GM [11].

Diet provides the nutritional supplies for life and growth, and some components exert valuable effects when consumed regularly. These components are called “functional foods” or “nutraceuticals” [12]. Consequently, functional foods contain bioactive substances, nutraceutics, which can be classified as micronutrients (vitamins and fatty acids) and nonnutrients (phytochemicals and probiotics) (see Table 1 in [13]). These components, with a wide range of chemical structures and functionality, provide different beneficial effects beyond simple nutrition, resulting in improved health.

Gut bacterial diversity is mainly affected by the diet, which may also affect its functional relationships with the host [14–17]. During their gastrointestinal passage, the components of the diet are metabolized by intestinal bacteria [18]. Diets rich in carbohydrates and simple sugars lead to *Firmicutes* and *Proteobacteria* proliferation, while those rich in saturated fat and animal protein favor *Bacteroidetes* and *Actinobacteria* [19]. Microbial diversity of the intestine decreases in diets with higher fat content [16]. Several physiological aspects of the gut environment can be influenced by the diet, then, including absorption of micronutrients, vitamins, and nutraceutics, and changes in pH of the gut environment, which in turn alters the balance of the GM [20]. Therefore, GM influences the biological activity of food compounds but is also a target for nutritional intervention to improve health [18].

On this basis, phytochemicals, like nutraceuticals that can influence GM, are being studied as coadjuvants to treat obesity and inflammatory diseases. In this review, we will present, organize, and discuss the most recent evidence that points to a relationship of the phytochemical effect on GM that affects obesity and/or inflammation, focusing on the effect of phytochemicals as potential prophylactics and candidates for the treatment of these diseases.

## 2. Phytochemicals Can Influence Obesity and Inflammatory Diseases through Affecting GM

Phytochemicals can be defined as “bioactive nonnutrient plant compounds present in fruits, vegetables, grains, and other plants, whose ingestion has been linked to reductions in the risk of major chronic diseases” [21]. Held to be phytochemicals, prebiotics are nondigestible food components (mainly carbohydrate polymers, such as fructooligosaccharides and mannooligosaccharides) that benefit the human body because they modulate GM through selective stimulation of some bacterial species proliferation in the colon, named “probiotics” [22]. These include endosymbionts such as lactic acid bacteria, bifidobacteria, yeast, and bacilli, which participate in the metabolism of their hosts [13]. Regarded as functional foods, both prebiotics and probiotics have been considered potential constituents of therapeutic interventions that modify GM in an attempt to modulate in turn some inflammatory diseases (comprehensively reviewed in [23]). On the other hand, the remaining phytochemical compounds may be classified on the basis of some common structural features into groups as follows: polyphenols and derivatives, carotenoids, and thiolsulfides, among others (see Table 1 in [13]). Of the latter, the polyphenols represent the largest group.

Polyphenols are secondary metabolites of plants and represent vastly diverse phytochemicals with complex chemical structures. They are commonly present in plant foods, such as cacao, coffee, dry legumes (seeds), fruits (like apples and berries), nuts, olives, some vegetables (such as lettuce and cabbage), tea, and wine. The daily intake of dietary phenols is estimated to be above 1 g, which is 10 times higher than the vitamin C intake from diet [24]. The interaction between polyphenols and GM has been well established [25]. Polyphenols are frequently conjugated as glycosides, which derive in aglycones when metabolized by GM. Generally, the intestinal metabolism of polyphenols includes hydrolysis of glycosides and esters, reduction of nonaromatic alkenes, and cleavage of the skeletons [26, 27]. Studies have reported that only a low number of polyphenols can be absorbed in the small intestine. The remaining (90–95%) nonabsorbed polyphenols reach the colon in high concentrations (up into the mM range), where they are degradated by microbial enzymes before their absorption [28]. Compared to their parent compounds, the permanence in plasma for metabolites is extended and they are finally eliminated in urine [29, 30]. GM, then, can regulate the health effects of polyphenols, and reciprocally, polyphenols can modulate GM and even interfere with its own bioavailability [31].

**Figure 1:**
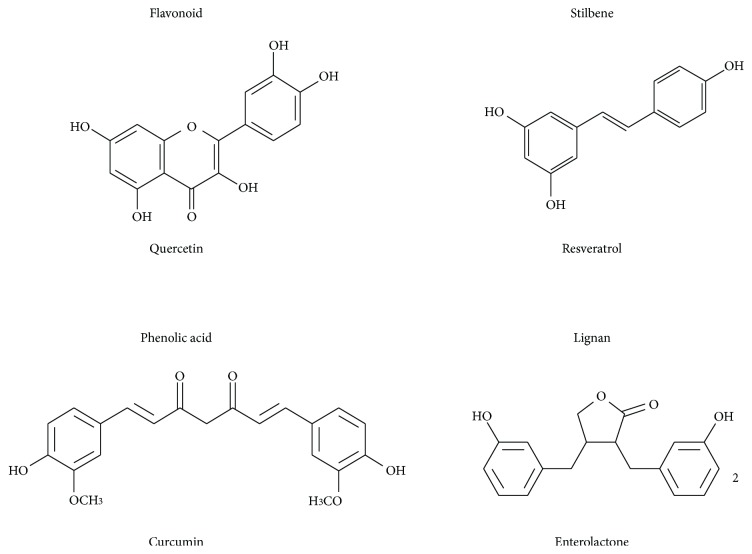
Chemical structure of representative molecules for the four main polyphenol groups.

Approximately 8000 structures of polyphenols have been identified [32], which can be classified into four main groups (Figure 1) as follows: (a) flavonoids (with eight subgroups), (b) phenolic acids (curcumin), (c) stilbenoids (resveratrol), and (d) lignanes. Polyphenols have been extensively studied over the past decade because of their strong antioxidant and anti-inflammatory properties and their possible role in the prevention and cotreatment of several chronic diseases, such as hypertension, diabetes, neurodegenerative diseases, and cancer [33–36]. In addition, polyphenols have recently attracted interest in the media and in the research community because of their potential role in reducing obesity, an increasingly serious health issue in different population age ranges [37, 38]. Polyphenols such as catechins, anthocyanins, curcumin, and resveratrol have been suggested as exerting beneficial effects on lipid and energy metabolism [39–41] and potentially on weight status. Multiple mechanisms of action have been proposed mostly as a result of animal and cell studies, such as inhibition of the differentiation of adipocytes [40], increased fatty acid oxidation [42], decreased fatty acid synthesis, increased thermogenesis, the facilitation of energy metabolism and weight management [43], and the inhibition of digestive enzymes [44].

Phenolic compounds from tea [45], wine [29], olives [46] and berries [47, 48] have demonstrated antimicrobial properties. Depending on their chemical structure, tea phenolics inhibit the growth of several bacterial species, such as *Bacteroides* spp., *Clostridium* spp., *Escherichia coli*, and *Salmonella typhimurium* [29]. Furthermore, tea catechins are able to change the mucin content of the ileum, affecting the bacterial adhesion and therefore their colonization [48]. Another study revealed that (+) catechin favored the growth of the *Clostridium coccoides*-*Eubacteriumrectale* group and *E. coli* but inhibited that of *Clostridium histolyticum*. In addition, the growth of beneficial bacteria, such as *Bifidobacterium* spp. and *Lactobacillus* spp., was nonaffected or even slightly favored [45, 49]. Both flavonoids and phenolic compounds reduce the adherence of *Lactobacillus rhamnosus* to intestinal epithelial cells [50]. The anthocyanins, a type of flavonoid, inhibit the growth of several pathogenic bacteria, including *Bacillus cereus*, *Helicobacter pylori*, *Salmonella* spp., and *Staphylococcus* spp. [47, 48]. Consequently, phytochemicals that affect the balance of the GM may influence obesity and inflammatory diseases.

Therefore, through the modulation of GM, polyphenols have the potential to generate health benefits. Although there is accumulative evidence concerning the polyphenolic effect on GM, the effects of the interaction between polyphenols and specific GM functions remain mostly uncharacterized; thus, much research remains to be conducted. We will focus on specific polyphenols that have been reported as able to affect GM and, in addition, influence obesity and/or inflammation.

## 3. Experimental Nutritional Interventions with Phytochemicals That Modify Gut Microbiota Exert an Effect on Obesity and/or Inflammatory Parameters

According to the United States National Agricultural Library, a “nutritional intervention” is “A clinical trial of diets or dietary supplements customized to one or more specific risk groups, such as cancer patients, pregnant women, Down syndrome children, populations with nutrient deficiencies, etc.” [51]. In a broader sense, we review herein the use of phytochemicals in experimental models (mainly polyphenols), which are able to modify GM and exert an effect on obesity and/or inflammatory parameters, in order to analyze and discuss their potential use for the prophylaxis and treatment of obesity and inflammatory diseases by the maintenance and control of GM.

**Figure 2:**
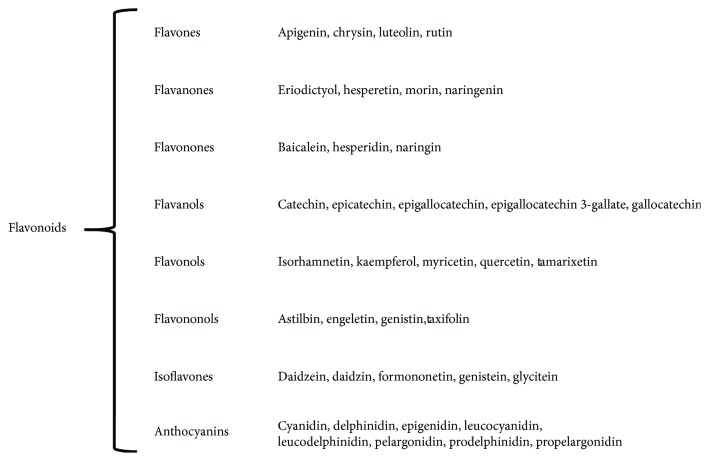
Classification of the eight foremost flavonoid subgroups.

To compile the information from scientific literature on the polyphenols that can be related with GM, we considered the following terms for search in PubMed: “gut microbiota” OR “intestinal microbiota” OR “gut flora” OR “intestinal flora” OR “gut microflora” OR “intestinal microflora,” and we added the specific compound (as listed in Figure 2). From this search, we can conclude that there is at least one report that correlates every polyphenol listed with GM. In addition, of the 40 listed compounds, there are 15 that yield at least 10 works that support the relationship between polyphenols and GM. However, there is still much work to be done in this area in terms of exploring in greater detail the specific actions of each compound on GM. Later, we added to these searches the following terms: “anti-inflammatory OR antiinflamatory” on one subsequent search, or “obesity” for another search. In both cases, the numbers of articles were scarce with a total of 116 and 71, respectively, although this number does not represent a real situation, because there are several articles that are repeated, and those that include more than one compound. From these articles, we extracted information that led to the indication of a relationship among the effects of different phytochemicals on the GM that affects obesity and/or the immune response (Table 1).

### 3.1. Flavonoids

The first and largest subgroup of polyphenols is integrated by flavonoids, with >6000 compounds identified and isolated from different plant sources [52], a large family of chemical compounds that constitutes plant and flower pigments and that shares the common function of being free radical scavengers. Due to the thousands of structurally different compounds, it becomes quite difficult to analyze all of them. Therefore, we performed a wide search of different specific compounds that have been reported in the literature and compiled them into eight subgroups, including the most representative compounds within each group (Figure 2). Essentially, all of these are widely recognized by their antioxidant [32, 53, 54] and anti-inflammatory [34, 55, 56] properties. Indeed, they inhibit reactive oxygen species (ROS) synthesis and hypoxia-signaling cascades, modulate cyclooxygenase 2 (COX-2), and block epidermal growth factor receptor (EGFR), insulin-like growth factor receptor-1 (IGFR-1), and nuclear factor-kappa B (NF-*κ*B) signaling pathways. In addition, flavonoids are able to modulate the angiogenic process [57], and the majority of these have been recently involved with obesity [58, 59].

#### 3.1.1. Flavones

Numerous studies have been undertaken on the influence of GM on the intestinal absorption and metabolism of particular flavones, such as apigenin, luteolin, and chrysin, both in rodents and in human cells [60–63]. On the other hand, there are multiple studies that associate different flavones with anti-inflammatory effects. This is the case for apigenin [64–67], luteolin [68, 69], and chrysin [34]. Furthermore, recent studies involve apigenin with the amelioration of obesity-related inflammation [70] and regulating lipid and glucose metabolism [71], luteolin with the amelioration of obesity-associated insulin resistance, hepatic steatosis and fat-diet-induced cognitive deficits [72–75], and chrysin, which inhibits peroxisome proliferator-activated receptor-*γ* (PPAR-*γ*) and CCAAT/enhancer binding protein A (C/EBP*α*), major adipogenic transcription factors in preadipocytes [75] and which also modulate enhanced lipid metabolism [76]. However, to the best of our knowledge, there is still no study that considers together these following three aspects: GM, inflammation, and obesity as positively affected by these flavones. Consequently, this constitutes a whole new avenue for studying these interactions.

#### 3.1.2. Flavanones

Like the previous subgroup, flavanones also influence and interact with GM [28, 77, 78]. The main compounds included here also exhibit anti-inflammatory properties, such as hesperetin [79, 80], naringenin [81], morin [82–84], and eriodictyol [85–87]. Additionally, they influence lipid metabolism as a potential preventive strategy for obesity. For instance, hesperetin exhibits lipid-lowering efficacy [88, 89]; naringenin regulates lipid and glucose metabolism [71] and also prevents hepatic steatosis and glucose intolerance [90] by suppressing macrophage infiltration into the adipose tissue [91]. In addition, both compounds improve membrane lipid composition [92]. Furthermore, morin exhibits antihyperlipidemic potential by reducing lipid accumulation [31, 93]. Finally, eriodictyol ameliorates lipid disorders and suppresses lipogenesis [94]. Taken together, all of this evidence strongly indicates that these compounds can be usefully applied to prevent or treat obesity and its associated inflammation, but it is relevant to take GM into account in order to incorporate it into the organism's metabolism. Again, there are to our knowledge no studies that correlate all three of these aspects.

#### 3.1.3. Flavonones

In this case, nomenclature represents a problem in the literature search, because the term “flavonones” is usually substituted by “flavanones,” which in fact represent a different subgroup. Due to this, compounds included in this subgroup were individually searched in databases. Three compounds were considered: hesperidin, naringin, and baicalein. In fact, the former two can be confused with similarly named compounds from the flavanone subgroup (see above) but constitute different compounds. As for all the polyphenols, the latter is metabolized by the GM [93, 95] and exhibits strong anti-inflammatory properties [79, 96, 97]. Additionally, these compounds also influence lipid metabolism as follows: hesperidin improves lipid metabolism against alcohol injury by reducing endoplasmic reticulum stress and DNA damage [98] and exhibits an antiobesity effect [99]; naringin also influences the lipid profile and ameliorates obesity [100], and finally, baicalein regulates early adipogenesis by inhibiting lipid accumulation and m-TOR signaling [101]. Again, there is a need for studies that take into account the following elements together, that is, GM metabolism of the polyphenols and their specific effect on lipid metabolism, obesity, and inflammation.

#### 3.1.4. Flavanols

This subgroup mainly comprises catechins, which are more abundant in the skin of fruits than in fruit pulp. Catechins found in cranberries may contribute to cancer prevention [102]. Catechins are abundant in green tea, to which has been attributed several beneficial impacts on health. Traditionally, green tea has been used to improve resistance to disease and to eliminate alcohol and toxins by clearing the urine and improve blood flow [103, 104]. Lately, emerging areas of interest have been the effects of green tea for the prevention of cancer and cardiovascular diseases, as well as their effects on angiogenesis, inflammation, and oxidation [105, 106].

This subgroup of flavonoids is one of the few that has been studied to date under the lens of their relationship with GM and their anti-inflammatory actions [107], as well as their role in lipid metabolism and obesity [105, 108]. Among the compounds included in this group, we find the following: catechin, epicatechin, epigallocatechin, epigallocatechin 3-gallate, and gallocatechin. Practically, all of these have already begun to be studied in the light of their relationship between GM and inflammation, as well as that related with lipid metabolism and obesity (see Table 1 for specific examples). However, much work remains to ascertain the mechanisms by which these compounds are able to benefit health.

#### 3.1.5. Flavonols

**Figure 3:**
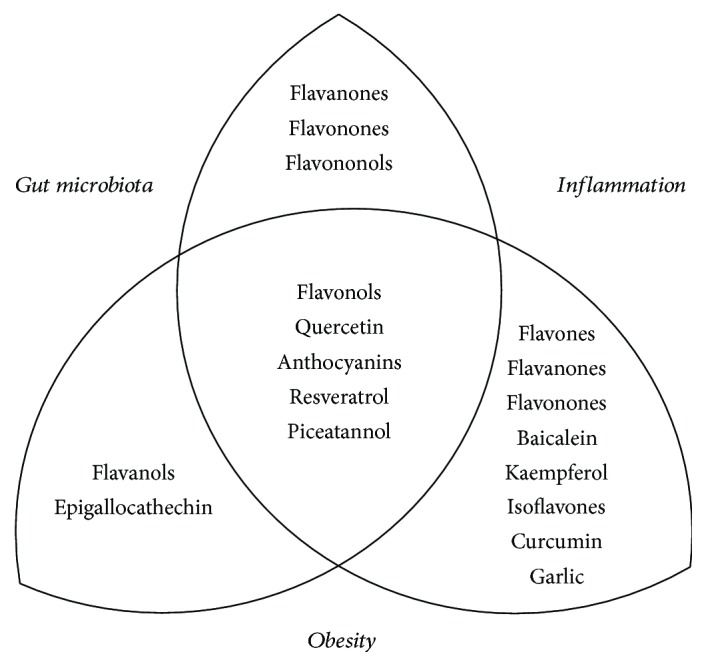
Phytochemicals that affect gut microbiota with anti-inflammatory and/or antiobesity properties.

Compounds in this subgroup have also been studied as related with GM and inflammation or obesity, mainly quercetin and kaempferol, while another three, rutin, myricetin, and isohamnetin, have not to our knowledge been studied within this context. Quercetin protects against high-fat diet-induced fatty liver disease by modulating GM imbalance and attenuating inflammation [109]. Kaempferol also exhibits protective properties, both anti-inflammatory and antioxidant, in adipocytes in response to proinflammatory stimuli [110]. These two works, by Porras et al., and Le Sage et al., respectively, constitute some clear examples of the experimental approximations that need to be done to increase our knowledge on the relationships already mentioned among phytochemicals, GM, inflammation, and obesity. Therefore, this subgroup constitutes that of the leading compounds in the study of the relationship among these three elements (Figure 3).

#### 3.1.6. Flavononols

This is another subgroup with nomenclature problems for the literature search, because the term “flavononols” is usually substituted by “flavonols,” which is a different group (see above). For this reason, compounds included in this group were individually searched. This subgroup includes genistein, taxifolin, engeletin, and astilbin. Again, all of these are metabolized by GM and also exhibit potent anti-inflammatory properties [111–114], as well as being able to influence energy metabolism (both lipid and carbohydrate) [115–117]. Despite this, to our knowledge there is a lack of research regarding the possible effects of this subgroup of flavonoids on obesity and/or inflammation through their effect on GM.

#### 3.1.7. Isoflavones

This subgroup has been partially studied with relation to GM and inflammation or obesity. It is made up of phytoestrogens, which are mainly present in soybeans. Isoflavones are metabolized by GM [30, 118, 119]. They also show an anti-inflammatory effect [120], as well as having had a hypocholesterolemic effect attributed to them [121]. The following are found included in this group: daidzein, genistein, glycitein, formononetin, and daidzin. Daidzein is metabolized by GM mainly into equol, which contributes to the beneficial effects of soybeans [122]; thus, it is relevant that dietary fat intake diminishes GM's ability to synthesize equol [123]. In addition, daidzein and genistein reduced lipid peroxidation *in vivo* and increased the resistance of low-density lipoproteins (LDL) to oxidation [124] and both exhibit an anti-inflammatory activity [125]. Glycitein affects gene expression in adipose tissue [126] and demonstrates antiobese and antidiabetic effects [127]. Additionally, together with daidzein and genistein, glycitein exhibits an anti-inflammatory and neuroprotective effect on microglial cells [128]. Finally, formononetin and daidzin have also received attention because of their anti-inflammatory properties [129–131]. Once again, this group would be interesting for further studies regarding their metabolism by GM in relation with inflammation and lipid metabolism for obesity.

#### 3.1.8. Anthocyanins

Anthocyanins are a class of flavonoids that are ubiquitously found in fruits and vegetables and they possess many pharmacological properties, for example, lipid-lowering, antioxidant, antiallergic, anti-inflammatory, antimicrobial, anticarcinogenic, and antidiabetic actions [132–135]. Strawberries constitute a source of anthocyanins and have been recently broadly evaluated for their effect on human health, due to their rich phytochemical content, effectiveness in rodent models, and almost no toxicity observed in pilot studies in humans [136, 137]. In rodent models, for example, strawberries have shown anticancer activity in several tissues [138]. This subgroup includes a long list of compounds, such as cyanidin, delphinidin, epigenidin, leucocyanidin, leucodelphinidin, pelargonidin, prodelphinidin, and propelargonidin. Although there are fewer than 70 papers that correlate at least one of these compounds with anti-inflammatory activity or obesity (or lipid metabolism), there are only a dozen papers, to our knowledge, which correlate any of these compounds with their metabolism by GM, and none of them associate this information among these aspects. Therefore, this constitutes a nearly complete virgin area still to be explored.

### 3.2. Phenolic Acids

#### 3.2.1. Curcumin

A second subgroup of polyphenols is constituted by phenolic acids, such as curcumin (diferuloylmethane), which is abundantly present in the rhizomes of the *Curcuma longa*, used both in traditional medicine and in cooking. Curcumin has been used for the coadjuvant treatment of a large diversity of diseases, including hepatic disorders, respiratory conditions, and inflammation and also obesity, diabetes, rheumatism, and even certain tumors. One relevant aspect to notice is that even at very high doses, no studies in animals or humans have revealed significant curcumin toxicity [139]. Curcumin possesses a great protective impact on acute alcoholic liver injury in mice and can improve the antioxidant activity of mice after acute administration of alcohol. It can increase the activity of antioxidant enzymes in liver tissues [140]. Curcumin is also metabolized by GM; the biotransformation of turmeric curcuminoids by human GM is reminiscent of equol production from the soybean isoflavone daidzein [141]. Curcumin modulates GM during colitis and colon cancer [142] and improves intestinal barrier function [141]. In addition, it is largely considered a potent anti-inflammatory and neuroprotective agent [143, 144], as well as a possible factor for the treatment of obesity [145–147]. The research on curcumin is extensive; notwithstanding, there are still very few papers that deal with the relationship of curcumin metabolism by GM, its action over intestinal permeability, and effect on obesity and/or inflammation (Table 1).

### 3.3. Stilbenes

#### 3.3.1. Resveratrol

The third subgroup of polyphenols comprises stilbenoids, such as resveratrol (3,5,4^'^-trihydroxystilbene) and piceatannol (3,3^'^,4,5^'^-trans-trihydroxystilbene). Resveratrol is a natural, nonflavonoid polyphenolic compound that can be found in grape wines, grape skins (red wine), pines, peanuts, mulberries, cranberries, and legumes, among other plant species, which synthesize it in response to stress or against pathogen invasion [148, 149]. Resveratrol is studied as a potent antioxidant with neuroprotective activity. Several *in vitro* and *in vivo* studies show various properties for resveratrol as a potent antioxidant and antiaging molecule, which also exhibits anti-inflammatory, cardioprotective, and anticancer effects, able to promote vascular endothelial function and enhance lipid metabolism [147, 150]. Principally, it is the anti-inflammatory effect of resveratrol which has been widely reported [151], as well as its antiobesity effect [152]. Regarding the GM effect, resveratrol favored the proliferation of *Bifidobacterium* and *Lactobacillus* and counteracts the virulence factors of *Proteus mirabilis* [29]. In fact, resveratrol exhibits pleiotropic actions, modulates transcription factor NF-*κ*B, and inhibits the cytochrome P450 isoenzyme CYP1 A1, as well as suppressing the expression and activity of cyclooxygenase enzymes, modulating p53, cyclins, and various phosphodiesterases, suppressing proinflammatory molecules, and inhibiting the expression of hypoxia-inducible transcription factor 1 (HIF-1*α*) and vascular endothelial growth factor (VEGF), among other actions [153]. Some studies analyze the effect of resveratrol on GM combined with their anti-inflammatory and antiobesity actions (Table 1). It constitutes a good example of the potential that the profound study of phytochemicals and their impact on health represents.

#### 3.3.2. Piceatannol

Piceatannol is a hydroxylated analogue of resveratrol found in various plants (mainly grapes and white tea). It is less studied than resveratrol but also exhibits a wide biological activity [154]. It mainly exhibits potent anticancer properties and also antioxidant and anti-inflammatory activities, which make it a potentially useful nutraceutical and possibly an attractive biomolecule for pharmacological use [59]. Recently, Hijona et al. [155] studied its beneficial effects on obesity. Although these are limited, it constitutes a promissory phytochemical molecule.

### 3.4. Organosulfur Compounds

#### 3.4.1. Garlic

In addition to polyphenols, another group of phytochemicals of relevance for health is the organosulfur compounds. For instance, garlic (*Allium sativum*) is a rich source of organosulfur compounds and exhibits a plethora of beneficial effects against microbial infections as well as cardioprotective, anticarcinogenic, and anti-inflammatory activity [156].

Nearly 80% of garlic's cysteine sulfoxide is constituted by alliin (allylcysteine sulfoxide). When raw or crushed garlic is chopped, the “allinase” enzyme is released which catalyzes sulfonic acid formation from cysteine sulfoxides and when the two react with each other, they produce an unstable compound: thiosulfinate or allicin. The *in vitro* breakdown of allicin produces numerous fat-soluble components: diallyl sulfide; DiAllylDiSulfide (DADS), and DiAllylTriSulfide (DATS). Likewise, vinyldithiins, S-allylcysteine, ajoene, S-1-prpenylcysteine, and S-allylmercaptocysteine are important constituents of garlic powder, oil, and extracts [157, 158].

Naturally occurring products have attracted the attention of researchers as sources of novel drugs and drug leads for the treatment of obesity [159–161]. *Allium* species have been used in herbolary or traditional medicine for the treatment of metabolic diseases, and *Allium*-derived extracts have recently become of interest for their antiobesity effects [162].

The chemical constituents of garlic are enzymes (asalliinase) and organosulfur compounds (such as alliin and its derived agent, allicin). The effect of garlic on different medical conditions (such as hypertension, hyperlipidemia, diabetes mellitus, rheumatic disease, the common cold, arteriosclerosis, and cancer) has been widely investigated. Garlic is known as a hypolipidemic agent because of its role in increasing the hydrolysis of triacylglycerols due to increased lipase activity [163]. Moreover, garlic reduces the biosynthesis of triacylglycerols through its blocking of nicotinamide adenine dinucleotide phosphate. On the other hand, garlic contains abundant antioxidants and can induce antioxidant enzymes [164]. Thus, garlic is a potential hepatoprotective agent against liver disorders [165]. Experimental studies have shown that garlic and its organosulfur compounds might reduce alcohol-related liver enzymes, glutathione reductase, alkaline phosphatase, lactate dehydrogenase, and alcohol dehydrogenase, as well as enhance liver antioxidant enzymes, and alleviate hepatic-fat accumulation [165–172]. However, there has been no clinical trial on patients with liver disorders [164].

## 4. Concluding Remarks and Perspectives

Several issues need to be solved before natural products can be effectively translated into the clinic. With regard to the best source of bioactive molecules or compounds, the following aspects should be considered: (a) if they are better acquired directly from food in the diet or from pharmacological sources (purified or through synthetic analogues) and (b) if they should be used alone or as a cotreatment in combination with approved drugs. Therefore, there is a need to develop specific clinical trials. Disadvantages of commercial nutraceutic preparations include the high variability in formulations (preparation methods and chemical composition), as well as the dosage quantification and the different means of administration. Research devoted to the optimization of phytochemical formulation and dosage has become of critical importance. Given the low bioavailability of phytochemicals, the development of more useful synthetic derivatives has become a great concern [173].

Once nutrients and nutraceuticals have been incorporated into the body, the gut environment is essential in maintaining homeostasis; in this sense, like GM, the surface of the intestinal mucous membrane plays a fundamental role in the preservation of homeostasis. Consequently, the correct functioning of its permeability is of great importance [174]. Several pathologies, as well as susceptibility to metabolic diseases, have been linked to alterations in the permeability of the intestinal barrier. Humans possess two interacting genomes: their own and that of their host microbiome, the majority of which resides in the gut, in the layer of mucin glycoproteins (mucus) produced by the cells called goblet cells [168]. The microbiome provides products such as vitamins and nutrients to host cells, thereby establishing a beneficial ecosystem for host physiology and preventing the arrival of pathogens [175]. Thus, a symbiotic relationship is established between both genomes, through the expression of pattern recognition receptors (PRRs) for the sense of the presence of intestinal microbiota, through the microbe-associated molecular patterns (MAMPs). This communication between the two genomes results in the accuracy of the mucosal barrier function, by regulating the production of its components: mucus, antimicrobial peptides, IgA and IL-22, facilitating homeostasis, and immune tolerance [175–177]. Therefore, GM and the human host influence each other by exchanging their metabolic active molecules [178], working together, as a hologenome, to maintain mutual health [179].

Another current challenge is convincing a skeptical health sector of the use of such compounds as medicines, or at least in conjunction with pharmaceutical medicines, which could serve both practitioners and patients better [180]. For instance, research on traditional Chinese medicine has substantially increased recently through the search for its molecular, cellular, and pharmacological bases, with the identification of active substances and the investigation of mechanisms of action [181]. Although the available cumulative data strongly suggest the positive effects of a large variety of phytochemicals in terms of health, it remains insufficient in order to directly extract solid conclusions, due mainly to the lack of confirmation, in human trials, of the results obtained by the animal model studies. Consequently, more research must be focused on the analysis of different phenolic compounds metabolized by GM and their influence on human health [182]. Results are crucial for the precise understanding of the influence of GM on the metabolism of micronutrients and phytochemicals within the human organism, and their metabolism undergone upon ingestion, in order to correctly attribute beneficial health properties to specific polyphenols with a more complete knowledge of their bioavailability, metabolism, and effects on carbohydrate and lipid metabolism, and therefore their use in treating obesity and inflammatory diseases.

## Figures and Tables

**Table 1:** Effects of different phytochemicals on GM and/or obesity with anti-inflammatory actions.

Phytochemicals	Compound	Model	Effect on gut microbiota	Antioxidant and anti-inflammatory effect	Effect on obesity	Ref
Polyphenols		C57BL/6 J ApcMin mice	Bacterial diversity was higher in the bilberry group than in the other groups	Attenuation of inflammation in cloudberry-fed mice		[183]
Flavonones	Baicalein	C57BL/6 J mice		Suppress activation of NF-*κ*B and decrease expression of iNOS and TGF-*β*	Activation of AMPK pathway and suppression of fatty acid synthesis, gluconeogenesis, and increased mitochondrial oxidation	[184]
Catechins	Epigallocatechin-3-gallate	C57BL/6 J mice	The *Firmicutes/Bacteroidetes* ratio is significantly lower in HFD + EGCG but higher in control diet + EGCG		Potential use for prevention, or therapy, for obesity-related and oxidative stress-induced health risks	[185]
	Epigallocatechin-3-gallate	C57BL/6 J mice	Regulates the dysbiosis and maintains the microbial ecology balance		Significant protective effect against obesity induced by high-fat diet (HFD)	[186]
	Epigallocatechin-3-gallate	Wistar rats	EGCG affects the growth of certain species of GM		Weights of abdominal adipose tissues fed 0.6% EGCG diet were suppressed. Regulated energy metabolism in the body	[187]
	Quercetin	C57BL/6 J mice	An increase in *Firmicutes*/*Bacteroidetes* ratio and in gram-negative bacteria and increased in *Helicobacter* by HFD. Quercetin treatment benefits GM balance	Quercetin reverted dysbiosis-mediated Toll-like receptor 4 (TLR-4) NF-*κ*B signaling pathway activation and related endotoxemia, with subsequent inhibition of inflammasome response and reticulum stress pathway activation	Benefits gut-liver axis activation associated to obesity, leading to the blockage of lipid metabolism gene expression deregulation	[109]
	Quercetin	Wistar rats	Quercetin supplementation attenuates *Firmicutes*/*Bacteroidetes* ratio and inhibiting the growth of bacterial species previously associated to diet-induced obesity (*Erysipelotrichaceae*, *Bacillus*, *Eubacterium cylindroides*). Quercetin was effective in lessening high-fat sucrose diet-induced GM dysbiosis			[188]
	Quercetin	Fischer 344 rats	Exerts prebiotic properties by decreased pH, increased butyrate production, and altered GM		Onion extract increased glutathione reductase (GR) and glutathione peroxidase (GPx1) activities in erythrocytes. In contrast, g-glutamate cysteine ligase catalytic subunit gene expression was upregulated	[189]
	Kaempferol	3 T3-L1 adipocytes		Kaempferol reduced LPS proinflammatory action. Demonstrating the anti-inflammatory and antioxidant effects	Concomitantly, polyphenols increased the production of adiponectin and PPAR*γ*, known as key anti-inflammatory and insulin-sensitizing mediators	[110]
Anthocyanins		C57BL/6 J mice	Feces of GM-deficient mice showed an increase in anthocyanins and a decrease in their phenolic acid metabolites, while a corresponding increase was observed in jejunum tissue		Mice with intact GM reduced body weight gain and improved glucose metabolism	[190]
Anthocyanins		C57BL/6 J mice		Anthocyanins could effectively reduce the expression levels of *IL-6* and *TNFα* genes, markedly increasing SOD and GPx activity	Anthocyanins reduced body weight could also reduce the size of adipocytes, leptin secretion, serum glucose, triglycerides, total cholesterol, LDL-cholesterol, and liver triglycerides	[191]
Phenolic acid	Curcumin	Mice	A direct effect of bioactive metabolites reaching the adipose tissue rather than from changes in GM composition	Nutritional doses of *Curcuma longa* is able to decrease proinflammatory cytokine expression in subcutaneous adipose tissue	An effect independent of adiposity, immune-cell recruitment, angiogenesis, or modulation of GM controlling inflammation	[192]
	Curcumin	LDLR−/− mice	Curcumin improves intestinal barrier function and prevents the development of metabolic diseases	Significantly attenuated the Western diet-induced increase in plasma LPS levels	Significantly reduced WD-induced glucose intolerance and atherosclerosis	[193]
	Curcumin	Human IEC lines Caco-2 and HT-29	Curcumin modulates chronic inflammatory diseases by reducing intestinal barrier dysfunction despite poor bioavailability	Curcumin significantly attenuated LPS-induced secretion of master cytokine IL-1*β* from IEC and macrophages. Also reduced IL-1*β*-induced activation of p38 MAPK in IEC and subsequent increase in expression of myosin light-chain kinase	Curcumin attenuates WD-induced development of type 2 diabetes mellitus and atherosclerosis	[194]
Stilbenes	Resveratrol	Kunming mice	HF microbiomes were clearly different from those in CT and HF-RES mice. After treatment, *Lactobacillus* and *Bifidobacterium* were significantly increased, whereas *Enterococcus faecalis* was significantly decreased, resulting in a higher abundance of *Bacteroidetes* and a lower abundance of *Firmicutes*		Treatment inhibited increases in body and fat weight in HF mice. Decreased blood glucose to control levels, decreased blood insulin and serum total cholesterol compared with HF mice. Severe steatosis seen in HF mice was well prevented in treated mice. Treatment significantly suppressed expression of PPAR-*γ*, Acc1, and Fas, suggesting inhibition of triglyceride storage in adipocytes	[195]
	Resveratrol	Glp1r−/− mice	Treatment modified GM	Decreased the inflammatory status of mice	Glucoregulatory action of RSV in HFD-fed diabetic wild-type mice, in part through modulation of the enteroendocrine axis *in vivo*	[196]
	Resveratrol	Wistar rats	Trans-resveratrol supplementation alone or in combination with quercetin scarcely modified the GM profile but acted at the intestinal level, altering mRNA expression of tight-junction proteins and inflammation-associated genes	Altering mRNA expression of tight-junction proteins and inflammation-associated genes	Administration of resveratrol and quercetin together prevented body weight gain and reduced serum insulin levels. Effectively reduced serum insulin levels and insulin resistance	[188]
	Resveratrol	Adipocytes		Generally, resveratrol opposed the effect induced by LPS, functioning as an ameliorating factor in disease state	LPS altering glycosylation processes of the cell. Resveratrol ameliorates dysfunctioning adipose tissue induced by inflammatory stimulation	[197]
	Resveratrol	Humans	Steroid metabolism of the affected GM should be studied in detail		Subtle but robust effects on several metabolic pathways	[198]
	Piceatannol	C57BL/6 mice	Pic altered the composition of the GM by increasing *Firmicutes* and *Lactobacillus* and decreasing *Bacteroidetes*		Pic significantly reduced mouse body weight in a dose-dependent manner. Significantly decreased the weight of liver, spleen, perigonadal, and retroperitoneal fat compared with the HFD group. Pic significantly reduced adipocyte cell size of perigonadal fat and decreased weight of liver	[199]
	Piceatannol	Zucker obese rats	It did not modify the profusion of the most abundant phyla in GM, though slight changes were observed in the abundance of several *Lactobacillus*, *Clostridium*, and *Bacteroides* species belonging to *Firmicutes* and *Bacteroidetes*	Shows a tendency to reduce plasma LPS by 30%	Pic did not reduce either hyperphagia or fat accumulation. There is a tendency toward the decrease of circulating on-esterified fatty acids, LDL-cholesterol, and lactate. While Pic tended to improve lipid handling, it did not mitigate hyperinsulinemia and cardiac hypertrophy	[155]
Organosulfur compounds	GEO (garlic essential oil) DADS (DiAllyl DiSulfide)	C57BL/6 mice		Significantly decreased the release of proinflammatory cytokines in liver, accompanied by elevated antioxidant capacity via inhibition of cytochrome P450 2E1 expression	GEO and DADS dose-dependently exerted antiobesity and antihyperlipidemic effects by reducing HFD-induced body weight gain, adipose tissue weight, and serum biochemical parameters	[200]
